# Anterior Knee Pain: State of the Art

**DOI:** 10.1186/s40798-022-00488-x

**Published:** 2022-07-30

**Authors:** Riccardo D’Ambrosi, Amit Meena, Akshya Raj, Nicola Ursino, Timothy E. Hewett

**Affiliations:** 1 IRCCS Orthopedic Institute Galeazzi, Via Galeazzi 4, 20161 Milan, Italy; 2 Dipartimento di Scienze Biomediche per la Salute, Università Degli Studi Di Milano, Milan, Italy; 3 Gelenkpunkt–Sports and Joint Surgery, Innsbruck, Austria; 4 Central Institute of Orthopaedics, Vardhman Mahavir Medical College and Safdarjung Hospital, New Delhi, India; 5 Hewett Global Consulting, Rochester, MN USA

**Keywords:** Anterior knee pain, Patella, Patellar instability, Patellofemoral pain syndrome

## Abstract

Anterior knee pain (AKP) is one of the most common conditions to bring active young patients to a sports injury clinic. It is a heterogeneous condition related to multiple causative factors. Compared to the general population, there appears to be a higher risk of development of patellofemoral osteoarthritis in patients with AKP. AKP can be detrimental to the patient’s quality of life and, in the larger context, significantly burdens the economy with high healthcare costs. This study aims to present a comprehensive evaluation of AKP to improve clinical daily practice. The causes of AKP can be traced not only to structures within and around the knee, but also to factors outside the knee, such as limb malalignment, weakness of specific hip muscle groups, and core and ligamentous laxity. Hence, AKP warrants a pointed evaluation of history and thorough clinical examination, complemented with relevant radiological investigations to identify its origin in the knee and its cause. Conservative management of the condition achieves good results in a majority of patients with AKP. Surgical management becomes necessary only when it is deemed to provide benefit—when the patient has well-characterized structural abnormalities of the knee or limb that correlate with the AKP clinically or in situations where the patient does not obtain significant or sustained relief from symptoms. AKP has a multifactorial etiology. The treatment strategy must be individualized to the patient based on the patient profile and specific cause identified. Hence, treatment of AKP warrants a pointed evaluation of history and thorough clinical examination complemented with relevant radiological investigations to identify the condition’s origin and its cause. A holistic approach focused on the patient as a whole will ensure a good clinical outcome, as much as a focus on the joint as the therapeutic target.

## Key Points

- Anterior knee pain is one of the most common conditions to bring active young patients to a sports injury clinic. It is a heterogeneous condition related to multiple causative factors.
- The causes of AKP can be traced not only to structures within and around the knee, but also to factors outside the knee, such as limb malalignment, weakness of specific hip muscle groups, and core and ligamentous laxity.
- Conservative management of the condition achieves good results in a majority of patients with AKP. Surgical management becomes necessary only when it is deemed to provide benefit—when the patient has well-characterized structural abnormalities of the knee or limb that correlate with the AKP clinically or in situations where the patient does not obtain significant or sustained relief from symptoms.

## Background and Relevance

Anterior knee pain (AKP) is one of the most common conditions to bring active young patients to a sports injury clinic; it is reported in about 40% of adolescent athletes [1].

Multiple studies have been conducted in specific population groups to quantify the incidence of AKP. Female military tactical athletes showed an incidence rate of 16.7 per 1000 person-years compared with enlisted males' incidence rate of 12.7 per 1000 person-years across all AKP diagnoses [2]. A recent study by Hannington et al. found that college basketball athletes in university and college basketball facilities presented a 60% incidence of AKP due to all causes [3]. Dey et al. reported the annual prevalence of patellofemoral pain (PFP) in the general population as 22.7% [4]. A recent systematic review by Smith et al. reported that females experience twice the risk of development of AKP due to patellofemoral causes than males [5]. The management of AKP has evolved over recent years. The condition was previously thought to be a self-limiting condition in most cases; the recent literature indicates the propensity of AKP toward repeated chronicity [6]. The management of AKP is aimed toward rehabilitation in most cases; however, recovery from patellofemoral AKP can be protracted, even despite patient adherence to the treatment and rehabilitation protocols [7].

The non-resolution of AKP results in varying levels of disability, which may preclude the patients’ participation in physical activity, sports, and work. The condition may even recur and persist for multiple years [8].

This chronic AKP not only becomes detrimental to the patients but also negatively impacts productivity due to absenteeism and has economic repercussions on the healthcare industry in accommodating the continued management of these patients. In the USA, the treatment of musculoskeletal pain disorders significantly burdens the economy, with an estimated annual cost of $213 billion in healthcare costs and sickness absence [9]. Patellofemoral AKP was found to be one of the most common causes of these costs and absences [10]. There appears to be a strong correlation between the severity of AKP and increased psychological impairment in patients. Machlachlan et al. found significantly higher levels of depression and catastrophization in cohorts with more severe patellofemoral AKP than in less severely AKP-impaired groups. The more severe groups also demonstrated significantly higher levels of kinesiophobia [11]. In addition, these patients may be at a higher risk of developing patellofemoral osteoarthritis (OA) than the general population. Overall, patients with patellofemoral AKP have been found to have poorer quality-of-life (QoL) scores compared to patients of the same age-group who do not have AKP and to the general population. The meta-analysis by Coburn et al. reported that individuals with PFP had poorer knee and health-related QoL scores than the pain-free controls and the general population [12].

Hence, AKP warrants a pointed evaluation of the patient’s history and a thorough clinical examination, complemented with relevant radiological investigations to identify the origin of AKP in the knee and its cause.

This study aims to present a comprehensive analysis of the evaluation of AKP pertaining to its diagnosis and selection of appropriate management strategies. AKP has a multifactorial etiology. The treatment strategy must be individualized to the patient depending upon the patient profile and specific cause identified.

## Diagnosis

### History-Taking

Thorough and meticulous history-taking is the foundation of an accurate diagnosis. One must pay attention to the patient’s symptoms and try to elicit them in as much detail as possible. Once the history is available to the clinician, further leading questions can be put forth to elaborate on the symptom complex. This will enable the clinician to formulate an accurate clinical diagnosis.

Patients with AKP can present with pain, swelling, instability, and functional impairment. Inquiries must be made about each symptom.

The onset of pain is insidious, and the progression is gradual—there may even be a waxing and waning pattern of symptoms. A history of trauma is uncommon, except in cases which may present post-traumatic patellar instability. It is crucial to know the circumstances in which the patellar dislocation occurred. If it took place after a minor trauma, there are probably one or more anatomical bony abnormalities in the knee joint. In this case, recurrent patellar dislocation is much more frequent.

The most prominent symptom, the pain experienced by the patient, must be evaluated on the basis of its location site. This is very important for localizing the pathology. For example, pain localized by the patient to the tibial tuberosity or inferior pole of patella may indicate a diagnosis of Osgood-Schlatter disease or Sinding-Larsen-Johansson disease, respectively.

Most patients, however, experience pain in one of two patterns: retro-patellar or peripatellar. Pain usually occurs in response to activities that burden the patellofemoral joint, such as climbing up or down stairs, squatting, kneeling, and prolonged flexion of the knee joint [13]. The so-called movie theater sign is observed when the patient experiences knee pain upon sitting with their knees flexed for a continuous period, such as while watching a movie in the theater. The pain may also emerge after long car drives. The pain may improve on knee extension. This points to a pathology of the extensor mechanism and not the femorotibial joint.

The patient usually describes the character of the pain as dull (sharp when there are exacerbations) and localizing in the patterns described above. Uncommonly, the pain may be diffuse and poorly localized but more prominent in the anterior aspect of the knee. The intensity of pain is variable and highly subjective, as is usually the case in painful musculoskeletal conditions.

There may be associated hyperalgesia, dysfunctional pain modulation that results from hyper-responsiveness of nociceptive neurons. Recent studies have identified the presence of signs of central sensitization in individuals with patellofemoral AKP [14].

Non-nociceptive stimuli may even evoke pain in some patients (allodynia). Psychological factors do play a role in pain and in pain modulation [15].

Post-traumatic cases will offer a history of painful knee swelling after the antecedent trauma. Other patients may provide a history of knee swelling that was resolved after a period of avoiding pain-provoking activity. There may be multiple episodes of knee joint swelling. Patients with instability describe a sensation of their knee joint “giving way” during walking which makes the patient apprehensive about walking up or down stairs or on uneven surfaces. The cause of the “giving way” sensation is related to reflex inhibition of the quadriceps. However, in chronic cases, the associated atrophy of the quadriceps may also contribute to this and must be observed in the clinical examination. Painless “giving way” can be associated with a transient patellar malposition or subluxation, a trapped synovial plica, an unstable cartilage flap, or any other ligamentous or meniscal instability of the femorotibial joint. Rotatory movements of the knee producing the “giving way” sensation are more commonly seen with anterior cruciate ligament injury or meniscal injuries rather than patellofemoral pathology [16].

These patients may report crepitus, although its significance in AKP is questionable and its presence is usually insignificant. A limitation of knee extension may be elicited in the history.

These patients must be questioned regarding specific activities that lead to the aggravation of their symptoms. Commonly, the appearance or worsening of the symptoms is related to the overuse of the knee, such as by repeatedly pressing the clutch pedal of the car during prolonged city driving. The pain might also be brought on by a new activity that the patient is not used to performing or increased performance of an accustomed activity [17]. Prompt identification of these activities of overuse with a thorough history and addressing them as part of the management is a key element of treating AKP.

There may be a component of lower-limb malalignment, and this must be explored during the clinical examination. Supra-physiological loading of the knee or physiological loading of a malaligned knee should both be kept in mind while eliciting the history [15–17].

As previously discussed, patients with more severe symptoms have been found to suffer from depression and catastrophization. They are also apprehensive about clinical examination maneuvers, that is, kinesiophobia. These symptoms should be sought while eliciting the patient’s personal history as they not only influence the symptoms but may hinder recovery and relief and may require earnest addressal.

Finally, any previous surgery on the symptomatic knee may give clues regarding the diagnosis; an iatrogenic patellofemoral instability can occur following surgery for deformity correction about the knee [15–17].

### Clinical Examination

**Fig. 1:**
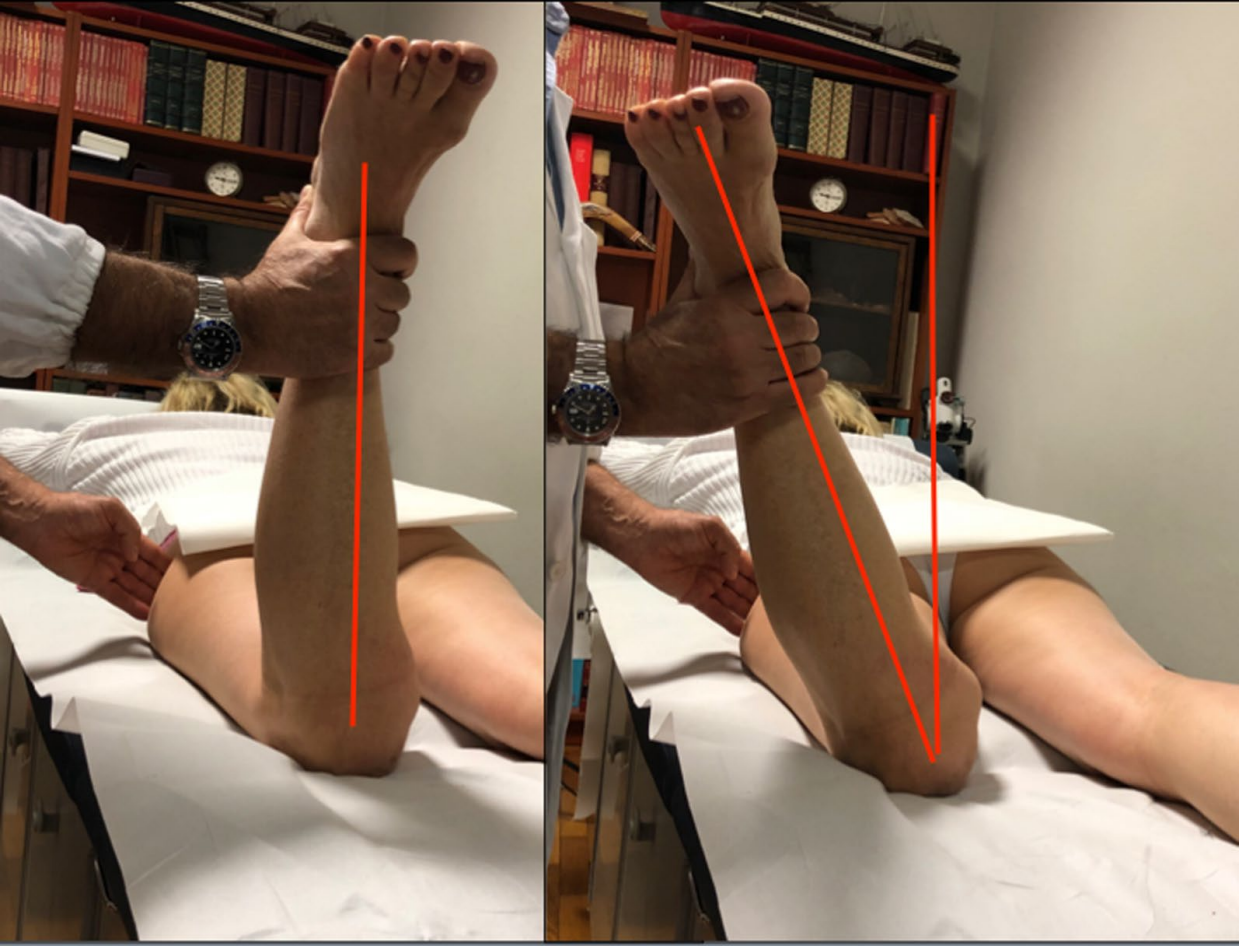
Craig's test is a passive test used to measure femoral anteversion or forward torsion of the femoral neck. The examiner palpates the greater trochanter and rotates the hip internally and externally until the greater trochanter lies at the lateral-most aspect of the hip (parallel to the examination table or bed), thereby projecting the femoral head into the center of the acetabulum. Interpretation: (1) Normal: At birth, the mean anteversion angle is 30°; it decreases to 8–15° in adults (angle of internal rotation). (2) Angle > 15°: Increased anteversion leads to squinting patellae and pigeon-toed walking (in-toeing), which is twice as common in girls. (3) Angle < 8°: Retroversion

**Fig. 2:**
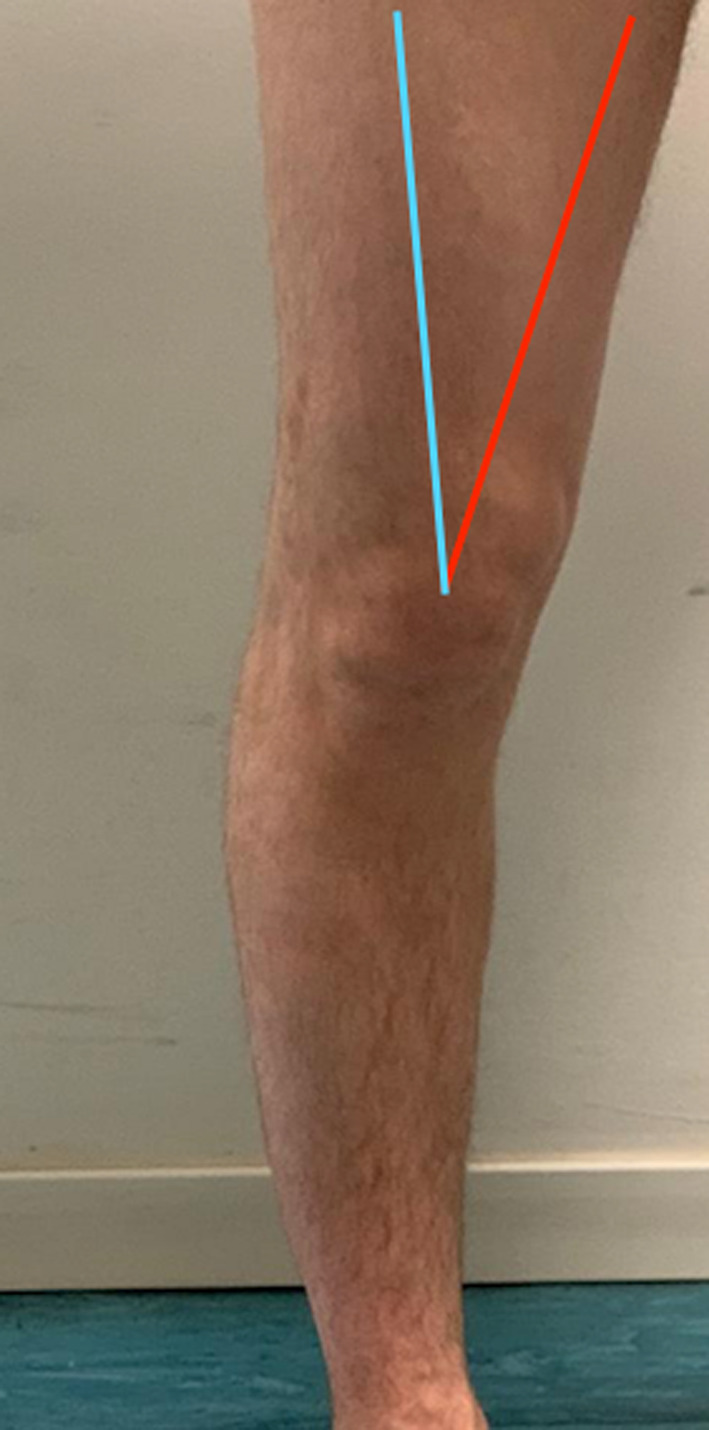
Q-angle (quadriceps angle) is the angle between the quadriceps tendon and the patellar tendon. It provides useful information about the knee joint’s alignment. The Q-angle is formed in the frontal plane by two line segments—one drawn from the anterior superior iliac spine (ASIS) to the center of the patella, and the other drawn from the center of the patella to the tibial tubercle. An increased Q-angle is a risk factor for patellar subluxation

A thorough and systematic physical examination is required to accurately diagnose the cause of AKP. The patient is made comfortable in a suitable environment for examination. Prior to any attempt to pinpoint the area of pain, it is wise to inspect the lower limb as a whole, especially with respect to its alignment, before moving on to the knee and patellar location, as this guides further examination. A genu valgum, excessive femoral anteversion, and consecutive tibial external rotation are independent risk factors for patellar instability. The femoral anteversion can be estimated by the Craig’s test performed with the patient prone with knees flexed to 90° (Fig. 1). A medial orientation of the patella, the so-called squinting patella, can be noted in the tibial external rotation, without associated excessive femoral anteversion. The Q-angle, which represents the vector of quadriceps pull, should be noted (Fig. 2). However, this may be spuriously normal in patients with lateral patellar subluxation, and the tubercle-sulcus angle at 90° knee flexion could be useful. The Q-angle is measured as the angle between the trans-epicondylar axis and a line joining the tibial tuberosity and the center of the patella with the knee flexed to 90° [18].

**Fig. 3:**
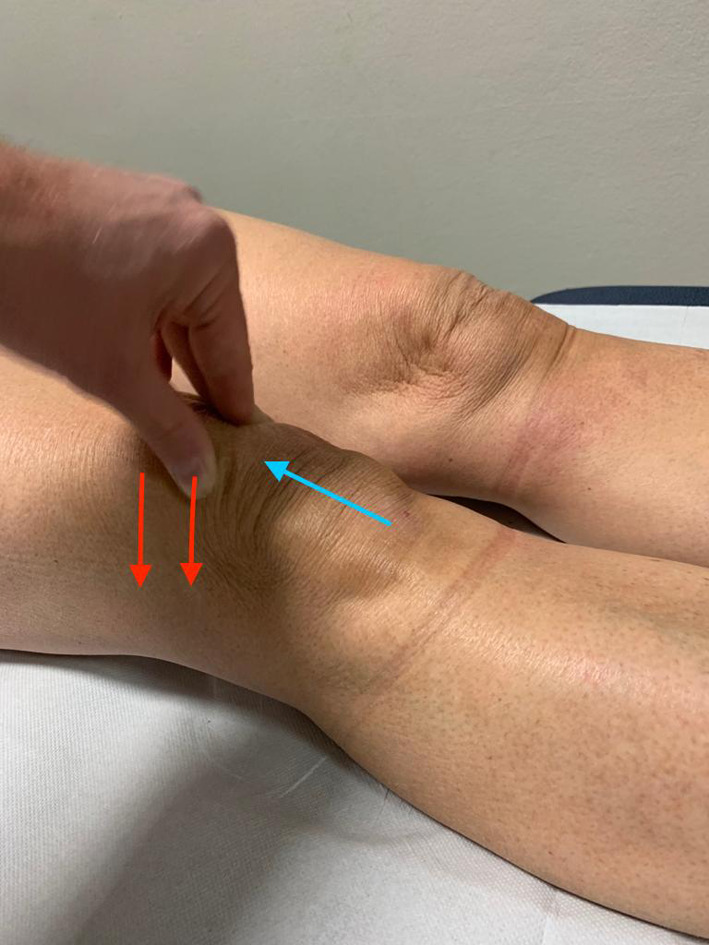
Hoffa's test for assessing fat pad tenderness. First, the knee is flexed. Next, the fat pad (lateral or medial) is depressed into the patellofemoral joint with the thumb. Next, the knee is extended while forcing the fat pad into the patellofemoral joint. A normal fat pad is not tender with this test. A painful, enlarged fat pad may be exquisitely tender with this maneuver

**Fig. 4:**
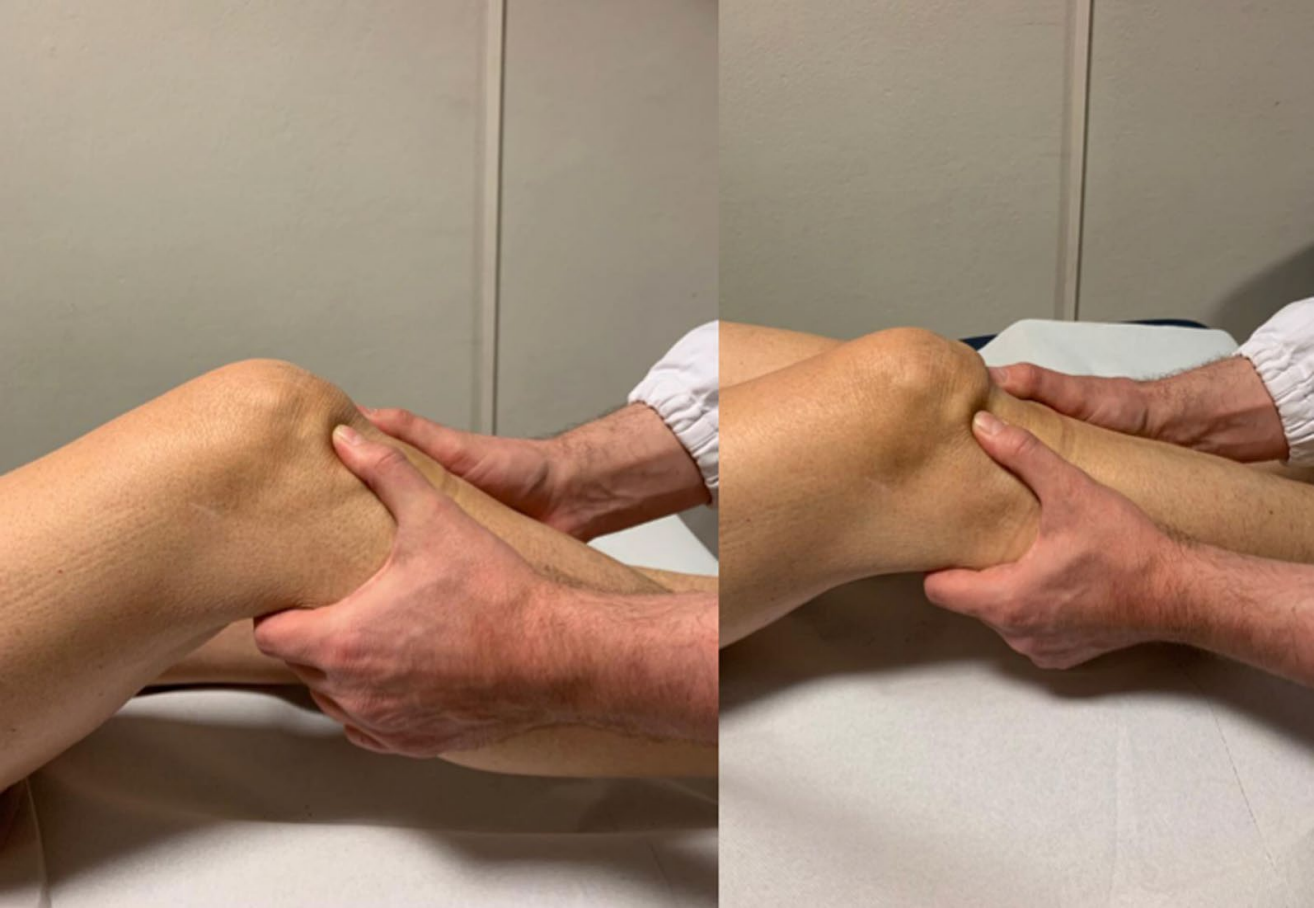
Patellofemoral grind test is used to determine patellofemoral syndrome. To perform this test, have the patient lie supine with the knee extended. Place the web space of your thumb on the superior border of the patient’s patella. Have the patient contract their quadriceps muscle while applying downward and inferior pressure on the patella. A positive test is signaled by pain with movement of the patella or an inability to complete the test

The next step is to localize the painful area to identify the injured or pathological structure, followed by palpation of the important patellofemoral and tibiofemoral landmarks. Tenderness of the distal patellar tip may indicate a patellar tendinopathy (Jumper’s knee), usually located more medially than laterally. Tenderness of the lateral–distal patellar tip is more often seen in the superolateral Hoffa impingement syndrome, associated with patella alta. Hoffa’s test is conducted to localize pain to the Hoffa fat pad, by exerting pressure on the fat pad with the fingers while the patient is asked to actively contract their quadriceps. In addition, the lateral retinaculum insertion to the patella is palpated. It is often painful in patellar maltracking, or after blunt trauma, associated with a hypomobile and tilted patella with a tight iliotibial tract. Lateral retinacular tightness is assessed by the patellar glide test and patellar tilt test. The patellar glide test assesses the medio-lateral translation of the patella in full extension of the knee. This should be less than half of the patellar width in normal cases. Excessive lateral translation of the patella indicates medial patellofemoral ligament (MPFL) insufficiency, while an excessive medial translation may be seen after a surgical release of the lateral retinaculum. Medio-lateral translation above 30° flexion of more than one-quarter the patellar width is highly associated with trochlear dysplasia. The patellar tilt test is conducted with the knee relaxed in extension and the lateral patellar margin lifted upwards. A limited upward (negative) tilt, where the patella cannot be lifted or tilted above the horizontal plane, indicates lateral retinacular tightness. The patellar grind test and finding of patellar facet tenderness indicate the origin of pain from the patellofemoral articular surfaces (Fig. 3). The patellar apprehension test is carried out with the patient in a supine position, and the knee flexed at 20–30°. The patella is pushed laterally, and the test is considered positive when the patient feels discomfort, pain, or avoidance with quadriceps activation, which pulls the patella back in place. Similarly, the cranio-caudal mobility of the patella may be checked and found decreased in cases of postoperative scarring of the Hoffa pad as well as in parapatellar retinacular scarring (Fig. 4) [18].

**Fig. 5:**
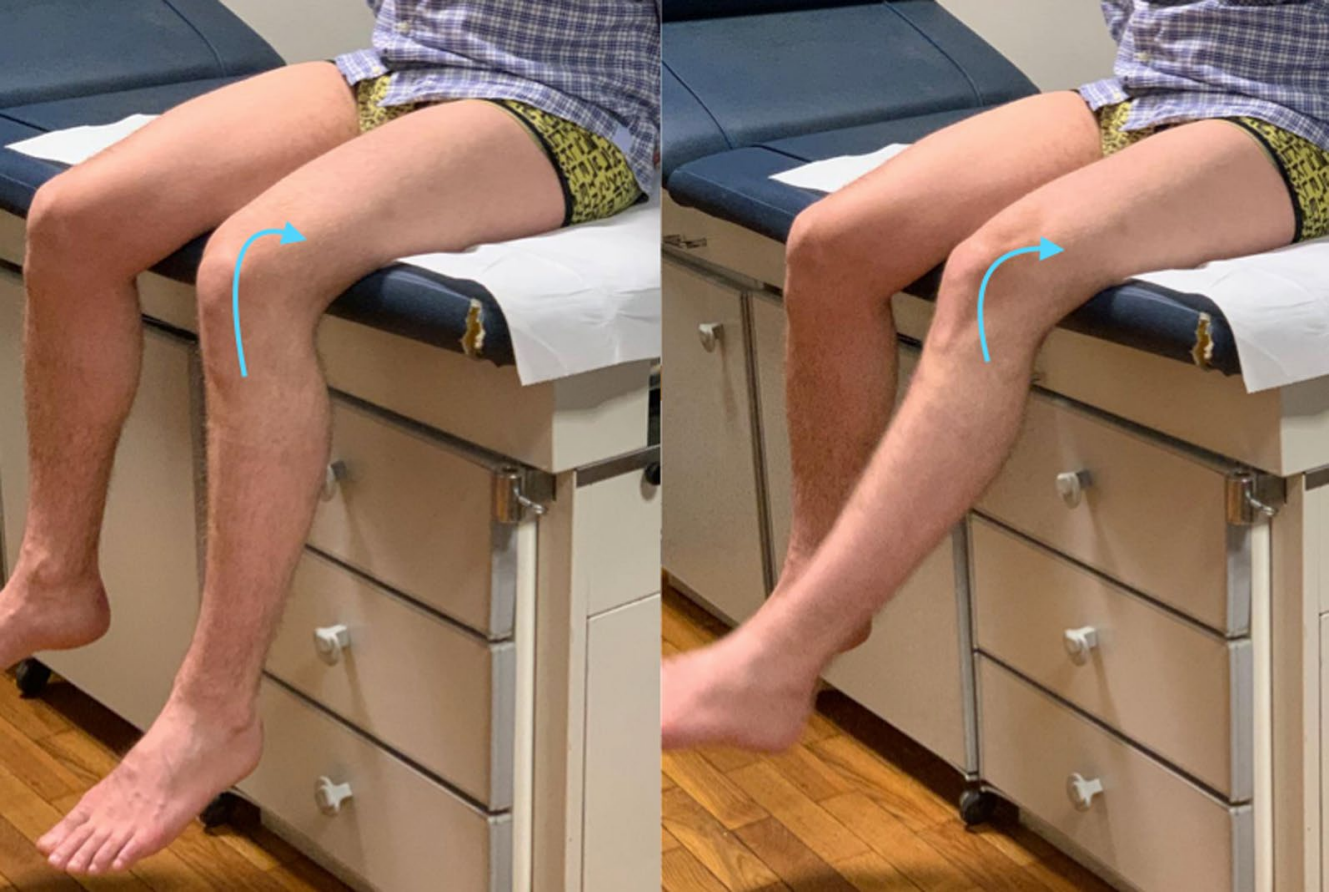
J-sign is a physical examination observation that correlates with poor patellar tracking. The patella takes an inverted J-shaped path as flexion is initiated from a fully extended position. It represents the engagement of the patella within the femoral trochlear groove as the knee flexes

In cases of malalignment, the patella jumps from lateral to medial into the trochlear groove during flexion. This is called the “J-sign” [16, 18] (Fig. 5).

**Fig. 6:**
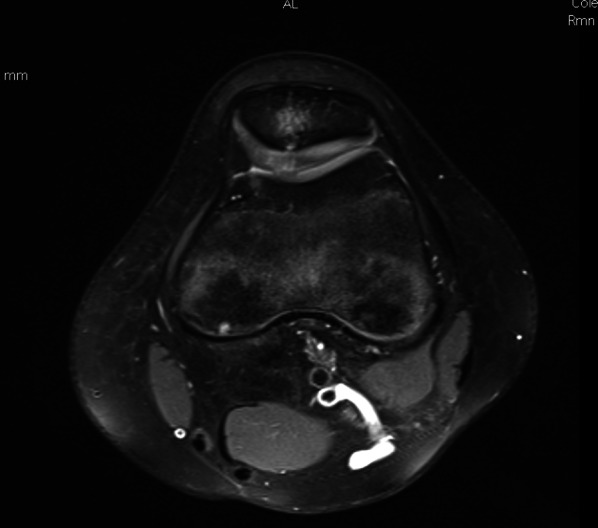
Left knee; MRI axial view; gross full-thickness erosion of the articular cartilage of the patellar ridge, with subchondral bone distress of an area of the patellar body characterized by edema with a small dystrophic cystic area. The picture overlaps with femoro-patellar dysplasia of the shallow femoral trochlea

In 90° flexion, one should also note the pes anserinus, as the aponeurosis of the sartorius muscle is in continuity with the medial parapatellar retinaculum and the superficial sheath of the aponeurosis of the vastus medialis. Tenderness may be elicited here after a patellar dislocation. A gap in the vastus medialis aponeurosis may also be appreciated on palpation. Tenderness at the femoral, or less commonly at the patellar, insertion of the medial MPFL can be elicited following a chronic or recurrent patellar dislocation. Previous scars should be palpated for tenderness [17].

The passive range and active range of motion of the symptomatic and the contralateral knees are measured. Indeed, it would be wise to measure the asymptomatic side first. Flexion contracture is ideally checked with the patient in a prone position. Restricted range of motion of the knee due to any postoperative stiffness tends to increase the patellofemoral contact pressure and cause AKP. Hip joints need to be examined for a torsional malalignment (if internal rotation is greater than external rotation of the hip). A hip pathology may result in pain radiating to the anterior knee. Generalized laxity must also be assessed via the Beighton score as this may contribute to patellofemoral instability [17, 18].

These patients with AKP tend to activate their quadriceps less, leading to a quadriceps avoidance gait pattern. There may be reduced strength of quadriceps extension strength, which has been shown to be predictive of AKP [18].

It becomes necessary to avoid maintaining focus solely on the knee joint. Hip abduction, hip extension, and hip external rotation weakness can be associated with AKP, as can core muscle weakness. The literature has shown that strengthening of hip musculature in patients with significant pain and less core endurance is more likely to have successful outcomes following hip and core muscle strengthening [19]. The single-leg squat or step-down test can assess gluteal function and the control of femoral rotation. Core function can be assessed by the bridge test—the patient lies supine, the knees are flexed to 90°, and the buttocks are lifted off the bed [20]. The strength of muscle groups can also be tested using a dynamometer.

## Imaging

The diagnosis of AKP is essentially a clinical one. Imaging plays a role in complementing the clinical examination by confirming the diagnosis and estimates the pathology quantitatively and qualitatively, ruling out others.

As always in orthopedic conditions, X-rays are the first step in imaging. Initially, the weight-bearing anteroposterior view, true lateral view, axial view, and skyline view of the patella are obtained. These views can show osseus morphological changes in the patellofemoral joint and knee joint. Of note is the measurement of the sulcus angle on the skyline view. An angle greater than 145°–150° is indicative of trochlear dysplasia. A systematic review by Paiva et al. identified 33 unique measurements used in trochlear dysplasia, especially recommending lateral trochlear inclination, the crossing sign, trochlear bump, trochlear depth, and ventral trochlear prominence as good-quality measurements [21].

When no significant abnormality is detected, or the patient’s symptoms are refractory, more detailed studies of the knee such as computed tomography (CT) and magnetic resonance imaging (MRI) are obtained. These enable estimation of radiological knee measurements, and any alterations in said measurements can be indicative of pathology.

On CT scans of the knee, in cases of PFP, tibial tuberosity–trochlear groove (TT–TG) distance is usually estimated. A distance of more than 21 mm of TT–TG is indicative of patellar maltracking. CT scans can be used to estimate torsional abnormalities of the lower limb, such as excessive femoral anteversion or external tibial torsion. Advantages of CT over plain radiography include its cross-sectional capability and ability to generate multi-planar reformations. CT is able to produce cross-sectional images in multiple planes, and this allows for a highly detailed evaluation of the patellar and trochlear morphology, patellofemoral relationship, and status of the joint. CT has many advantages over MRI including being cheaper, quicker, able to accommodate larger patients due to a larger gantry, and no contraindications for implants. CT is the best modality to delineate bony pathology. Three-dimensional CT images are also very helpful in surgical planning. The disadvantages of CT compared to MRI include the use of ionizing radiation, which reduces soft tissue resolution [22].

MRI of the knee is currently used extensively for evaluation of AKP and is a vital tool for evaluation of the potential cause(s) due to the complex structure and biomechanics of the knee (Table 1) [22].

**Table 1:** MRI checklist for the assessment of patellar maltracking

Patellar maltracking: associated features	Methods of assessment (among others)	Significance
Trochlear dysplasia	Trochlear depth, lateral trochlear inclination, trochlear facet asymmetry (evaluated on most cranial axial images showing cartilage, approximately 3 cm above the joint line)	Geometric abnormality of the trochlear groove that can result in abnormal tracking of the patella along the trochlea
Patella alta	Insall–Salvati index, Caton–Deschamps index	Relates to a long patellar tendon. For the patella to engage with the trochlea, a higher degree of flexion than normal is needed
Lateralization of the tibial tuberosity	Tibial tubercle–trochlear groove distance (TT–TG)	High TT–TG would exert lateral pressure on the patella during extension and, if not counteracted by vastus medialis contraction, may predispose to patellar subluxation
Lateral patellar tilt	Patellar tilt angle, patellofemoral angle	Sensitive marker for patellar instability present in significant proportion of patients
Lateral patellar tilt	Edema at the superolateral aspect of Hoffa’s fat pad on MRI	Sensitive marker for patellar instability present in significant proportion of patients
Hoffa’s fat pad impingement	Edema at the superolateral aspect of Hoffa’s fat pad on MRI	Significant association with several patellar maltracking indicators
MPFL and medial patellar retinacular injury	Best evaluated on the axial fluid-sensitive MRI sequence	Present in the majority of patellar dislocation cases
Chondral and osteochondral damage	MRI can show discrete osteochondral defect or various degrees of patellofemoral cartilage loss (Fig. 6)	Patellar maltracking is a significant risk factor for patellofemoral OA. Patellar dislocation can result in discrete osteochondral defects at the patella or lateral femoral condyle

The TT–TG distance can also be evaluated on MRI. The TT–TG is measured as the distance from the anterior most point of the tibial tuberosity and the deepest point on the femoral trochlea, by taking two lines perpendicular to a tangential line to the posterior borders of the femoral condyles. The TT–TG distance decreases with knee flexion and reduces with weight-bearing [22].

Determining the deepest part of the trochlear groove can be difficult when assessing the TT–TTG in the presence of trochlear dysplasia; therefore, an alternative method for assessment of tibial tubercle position was proposed, which measured the distance in reference to the posterior cruciate ligament rather than the trochlea (tibial tubercle–posterior cruciate ligament distance [TT–PCL]), with a proposed pathological threshold of 21 mm [23, 24].

The patellar retinaculum and MPFL can be seen on MRI as well-defined low-signal-intensity bands. The MPFL is best seen on an axial MRI on the slice just distal to the vastus medialis oblique (VMO). On T2-weighted MR images, a sprain is depicted as the thickening of the retinaculum with signal intensity signifying edema and hemorrhage. Complete disruption and avulsion are seen as a discontinuity of ligament fibers with associated edema [22–24].

**Fig. 7:**
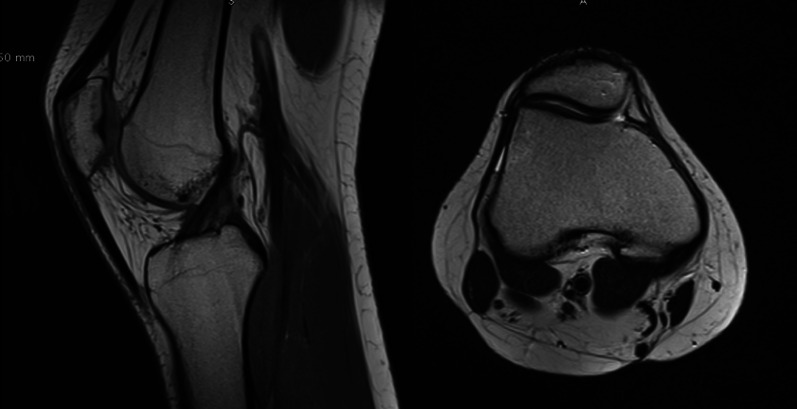
Right knee; MRI sagittal and axial view; patella alta (Insall–Salvati ratio 1.9) in modest external tilt in the presence of dysplasia by the shallow femoral trochlea

MRI features of patellar tendinitis include focal thickening of the proximal one-third of the tendon, an AP diameter greater than 7 mm, and focal T2 hyperintensity within the proximal tendon, most commonly involving the medial one-third of the tendon; this may extend to involve the central third of the tendon as well. Other imaging findings include an indistinct posterior tendon border and edema in the adjacent Hoffa’s fat pad [25] (Fig. 7).

Osgood-Schlatter disease shows an enlarged patellar tendon with T1 and T2 hyperintensity at its insertion on the tibial tuberosity indicative of tendinosis or tendinitis. Edema of the deep infrapatellar bursa with surrounding soft tissue edema may be appreciated along with marrow edema evident in the region of the tibial tuberosity. In Sinding-Larsen-Johansson (SLJ) syndrome, the MRI shows an osseous avulsion injury at the proximal patellar insertion of the ligamentum patellae without much injury to the cartilage. This distinguishes SLJ from a patellar sleeve avulsion. It is important to distinguish between the two as they may require different treatment strategies, with conservative management for SLJ versus possible internal fixation for displaced patellar sleeve avulsion fractures.

The medio-patellar plica is the most likely plica to cause symptoms when it becomes inflamed, thickened, and fibrotic. On MRI, the normal plica has low signal on both T1- and T2-weighted images and is easily identified with some degree of joint distention. Symptomatic synovial plicae will be seen to have hyperintensities on T2 sequence. When the articular fluid is not enough to delineate the plica, MR arthrography is a useful technique to better delineate it. The medio-patellar plica has been classified into four types—A through D—based on size and extent of coverage over the anterior surface of the medial femoral condyle. Types C and D can be trapped between the medial condyle and the patella, become thickened, and even cause chondral damage [24, 25].

A quadriceps tendon (QT) tear is the second-most common injury to the extensor mechanism after patellar fracture. It occurs most commonly at the tendo-osseous junction at the patellar base and can be caused by recalcitrant AKP with weak knee extension [26]. On MRI, hematoma and surrounding edema at the site of injury manifests as an area of increased signal intensity on T2-weighted images. Partial tendon tear can be appreciated well on axial T2/STIR sequences. In a complete quadriceps tendon tear, it is important to note the separation of torn edges, the characteristics of torn edges of the tendon, and the presence of hematoma, as these may influence surgical planning.

## Conservative Treatment

AKP has a characteristically multifactorial etiological profile, and the myriad causes each have to be treated based on the symptoms and physical examination findings, while aiming to unload the stresses on the patellofemoral joint and peri-articular soft tissues. A weak quadriceps has been shown to not only contribute to increased joint stresses, but also increase the risk of cartilage loss in the patellofemoral and tibiofemoral compartments [27]. There is now evidence to indicate that quadriceps weakness may be more significant in progression of patellofemoral arthritis in women. The Multicenter Osteoarthritis Study by Culvenor et al. found that quadriceps weakness increased the risk of worsening lateral patellofemoral cartilage damage in women, indicating that optimizing quadriceps strength may help prevent worsening of structural damage in women’s patellofemoral joints [28].

Therefore, it appears worthwhile to initiate the patient on a program for strengthening the quadriceps, particularly the VMO. Taping combined with strengthening of the VMO seems to offer better results [29]; it has also been shown to have a role in improvement in patellofemoral biomechanics. Venkatapathy et al. found that isometric quadriceps activation and vastus medialis oblique strengthening can reduce the Q-angle significantly [30]. This will improve the seating of the patella in the trochlea and optimize the lower limb mechanics, which should decrease the patient’s symptoms.

Taping can move the inferior pole of the patella away from the inflamed Hoffa’s fat pad. When combined with activity modification to reduce the duration the knee is kept in flexion, taping can provide significant pain relief.

## Surgical Treatment

Although the primary treatment of AKP is non-surgical, there may be situations where the patient does not obtain significant or sustained relief from symptoms. In certain cases, like limb malalignment or trochlear dysplasia, the dysfunction caused by the structural abnormality may warrant surgical correction if one aims to achieve optimum knee and lower limb mechanics, in order to allay the patient’s symptoms.

### Synovial Plicae

A painful plica can be localized by palpation of the knee region where the patient indicates their pain originates from. Most commonly, the plica can be felt snapping underneath the palpating fingers in the medial infrapatellar region. When the AKP is diagnosed as occurring due to an inflamed and hypertrophied synovial plica, the appropriate management is arthroscopic resection. There may be chronic cases where there is synovial hypertrophy, particularly at the inferior pole of the patella, which might warrant a synovectomy using a radiofrequencies probe [31].

### Patellofemoral Cartilage Repair

Various procedures have been described for patellofemoral chondral lesions, viz. debridement/chondroplasty, bone marrow stimulation, autologous chondrocyte implantation (ACI), osteochondral autograft transfer (OAT), and osteochondral allograft transplantation (OCA).

Chondroplasty, also referred to as cartilage debridement, aims to transform an irregular and unstable cartilage lesion into a more regular and stable construct. It is typically indicated for partial or full-thickness chondral lesions smaller than 1–2 cm^2^ but can also be performed in larger lesions, preparing the site for a subsequent cartilage repair procedure such as ACI [32].

Bone marrow stimulation techniques induce the migration mesenchymal stem cells from the marrow to the cartilage defect and promote fibrocartilaginous healing to fill it up. The technique most often used is the microfracture technique, using angulated awls. This method is usually used for defects of 2 cm^2^ but can sometimes be used in defects up to 4 cm^2^. Another method is the Pridie drilling technique. However, these techniques have been less effective on the patella.

A recent technique is the bone marrow aspirate concentrate (BMAC) where clotted bone marrow is placed in the chondral defect beneath the membrane, to stimulate healing at the defect. This can be used as an adjuvant method to OAT or ACI.

ACI is currently performed as a two-stage procedure [33]. It entails arthroscopic harvesting of 100–300 mg of full-thickness articular cartilage from non-weight-bearing areas of the femoral condyle, or the superior and lateral margin of the intercondylar notch; in vitro processing and culture of chondrocytes for two weeks; and implantation into the debrided cartilaginous defect in a second stage. The debridement must be performed carefully to avoid damage to the subchondral plate and create a stable vertical wall. The original method used a periosteal patch; second-generation techniques used collagen membranes. In the current third-generation ACI, also known as matrix-induced autologous chondrocyte implantation (MACI) [34], the chondrocytes are cultured in vitro but replicated inside a three-dimensional scaffold membrane, which simplifies the procedure and obviates the need for chondrocyte injection and watertight closure of the membrane. An ACI can be performed for full-thickness articular cartilage lesions greater than 3 cm^2^.

Osteochondral autografts can be used to resurface painful articular lesions. Donor site morbidity remains a concern in OAT procedures and limits the indication for OAT to only small cartilage lesions up to 2–3 cm^2^ in size [35]. Allograft transfers may be considered for larger femoral-sided defects of the patellofemoral joint.

### Tibial Tuberosity Transfers

Malalignment of the extensor mechanism can be treated with an anteriomedialization of the tibial tuberosity. The diagnosis may be made on clinical grounds using the Q-angle. However, the TT–TG distance on CT scans is the most accurate measurement related to the malaligned pull of the quadriceps. The goal of tibial tubercle transfer is to reduce the TT–TG distance to between 10 and 15 mm. It was introduced for patellofemoral instability [36]. However, this procedure can improve patellofemoral contact mechanics in patients with patellofemoral malalignment by redirecting contact forces from caudal to cranial and from lateral to medial on the patellar articular surface.

Due consideration should be given to the trochlear shape and depth; the deeper the trochlea, the greater the chance of over-medialization. This may cause impingement of the medial patellar facet and, hence, pain.

Anteriomedialization of the tibial tuberosity is usually combined with cartilage repair procedures to address both malalignment and its sequel of patellofemoral osteochondral lesions.

### Trochleoplasty

Lateral-facet elevation trochleoplasty is indicated in patients with a flat or shallow trochlea, without trochlear prominence and any other significant factors of instability. Care must be taken to ensure that the procedure does not cause greater trochlear prominence, which might give rise to impingement in flexion. The sulcus-deepening trochleoplasty was first described by Masse in 1978 [37]; it was subsequently modified by Dejour in 1987 [38].

Sulcus-deepening trochleoplasty is more etiology-based and is indicated in severe (type B or D) dysplasia, in which the trochlea is prominent and the patella impinges on it. The best indication is of a patient who has an abnormal patellar tracking with a J-sign. The deepening trochleoplasty is like a proximal realignment and decreases the TT–TG. Moreover, it is not always necessary to add a distal realignment. One can combine this procedure with soft tissue surgery, such as a medial patellofemoral ligament reconstruction. Although more challenging to perform, this trochleoplasty has the advantage that it will address the root cause of the patellar dislocation by directly addressing the pathoanatomy of the different grades of trochlear dysplasia.

### Femoral and Tibial Osteotomies

In a small number of cases, patellar instability may be due to lower-limb malalignment, particularly excessive valgus or torsion. Valgus knee deformity causes an increased lateral vector to the quadriceps pull, which predisposes to a lateral patellar subluxation. Valgus malalignment of the knee of more than 10 degrees is to be taken as pathological. The problem arises on the femoral side and is due to a hypoplastic lateral condyle. Correction of genu valgum due to a hypoplastic lateral femoral condyle is achieved by a medial closing wedge osteotomy of the distal femur. Torsional deformities will exhibit a compound pattern of excessive femoral anteversion and excessive external torsion of the tibia. When making the decision to operate, the surgeon should bear in mind that osteotomy is a major procedure to correct what is often a well-tolerated condition. Ideally, the intertrochanteric region is where femoral derotation should be performed. It is best to perform tibial derotation just proximal to the tibial tuberosity [39].

### Arthroplasty

**Fig. 8:**
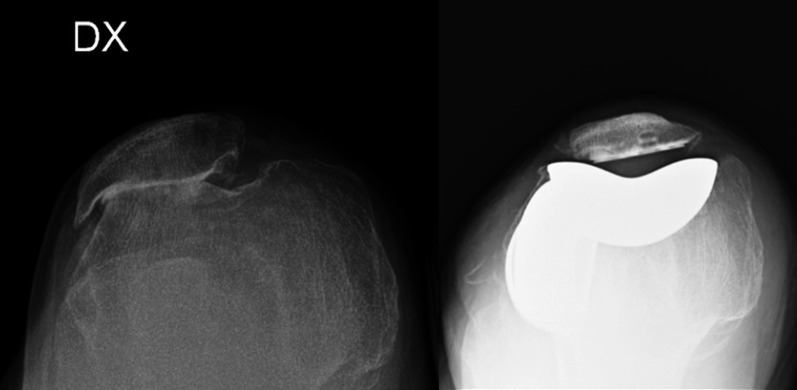
Axial right  knee radiographs showing a high degree of osteoarthritis of the patellofemoral joint, with abolition of the joint line treated with isolated patellofemoral arthroplasty

Patellofemoral arthroplasty may become necessary, particularly in elderly patients, with advanced patellofemoral arthritis and gross destruction of the joint [40] (Fig. 8).

## Conclusion

**Fig. 9:**
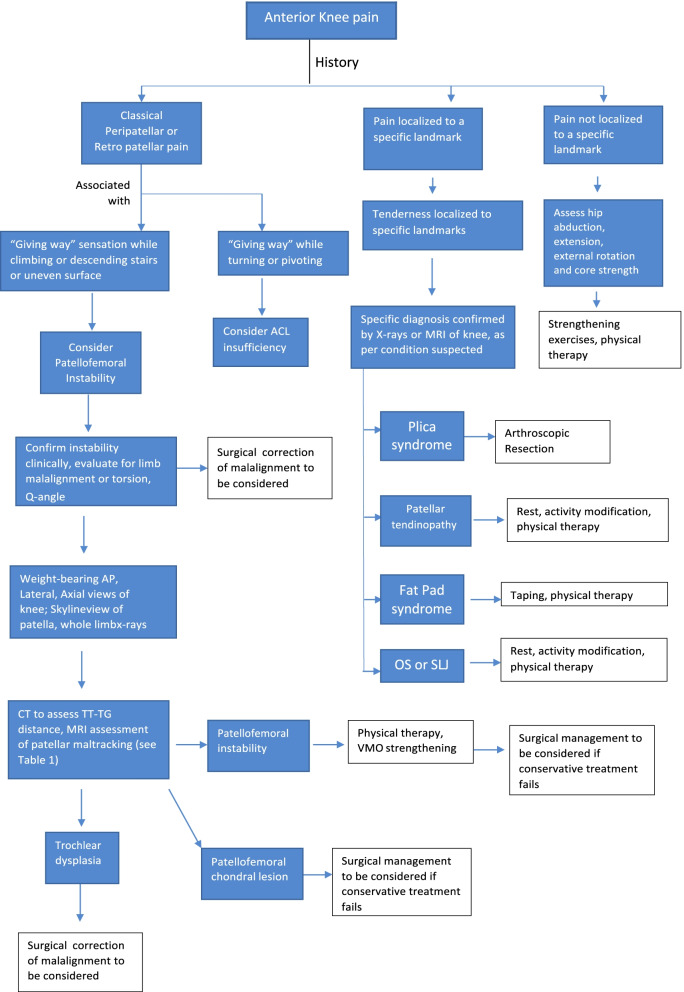
Proposed algorithm treatment based on the current literature. *MRI* Magnetic resonance imaging, *ACL* anterior cruciate ligament, *AP* anteroposterior, *CT* computed tomography, *TT-TG* tibial tuberosity–trochlear groove distance, *OS* Osgood-Schlatter disease, *SLJ* Sinding-Larsen-Johansson disease

Although the tendency is to consider AKP as a single entity in clinical practice, it is rather diverse in its etiology. The ultimate manifestation of pain is the result of varied predisposing factors and pathoanatomy. The various pathologies must be kept in mind during evaluation of the patient in order to accurately localize the cause. Once the cause is identified, the patient must be given an explanation regarding their condition, how it has occurred, and how they can deal with it; this will go a long way toward allaying their apprehensions. Indeed, it has been observed that, in addition to anatomical and biomechanical factors, psychological and psychosocial factors might play a more significant role than previously considered. Hence, it becomes imperative to assess the patient profile and tailor the management on an individual basis in order to achieve the best outcome. The severity of AKP has strong associations with depression and anxiety [11]. Catastrophization represents a significant barrier to a good clinical outcome in such patients. A holistic approach focused on the patient as a whole, as much as a focus on the joint as the therapeutic target, is hence warranted. Forthcoming biological methods and the continued improved understanding of AKP should help clinicians to provide better outcomes in the future. A treatment algorithm is proposed to improve clinical daily practice and to ensure better treatment possibilities for conditions ranging from simple AKP to advanced patellofemoral osteoarthritis (Fig. 9).

## Data Availability

All data are available upon request to the corresponding author.
